# Influence of Corrosion-Inhibiting Monolayers on the Bond Strength and Durability of Reinforced Concrete Structures Under Service Conditions

**DOI:** 10.3390/ma18071656

**Published:** 2025-04-04

**Authors:** Pablo Monzón-Bello, Roberto Vengut-Tro, Juan Soto-Camino, Manuel Octavio Valcuende-Payá

**Affiliations:** 1 Department of Architectural Construction, Universitat Politècnica de València, Camino de Vera s/n, E-46022 Valencia, Spain; mvalcuen@csa.upv.es; 2 Institute of Molecular Recognition and Technological Development (IDM), Universitat Politècnica de València, Camino de Vera s/n, E-46022 Valencia, Spain; roventr@etsid.upv.es (R.V.-T.); jsotoca@upv.es (J.S.-C.)

**Keywords:** reinforced concrete, corrosion inhibitors, bond strength, corrosion rate

## Abstract

Corrosion protection in reinforced concrete structures exposed to aggressive environments remains a critical challenge in civil and architectural engineering. One promising approach involves the application of corrosion-inhibiting monolayers on the reinforcement, such as those formed using 4-aminobenzoic acid. Two methods have previously been employed to generate these monolayers: one relying on the adhesion of an organic compound and the other utilising an externally modified approach via electrolysis. This study assesses the influence of this treatment on the steel–concrete bond strength and durability, both critical properties for the structural performance of reinforced concrete under service conditions. For this purpose, pull-out tests were performed on specimens subjected to load–unload cycles to analyse bond behaviour and monolayer integrity. The results indicate that these treatments do not adversely affect the bond strength between reinforcement and concrete. Furthermore, the rebars treated with the inhibitor exhibit less corrosion damage than the untreated rebars. This fact is particularly significant in the rebars treated using the natural adhesion method, with the steel section loss being 32–37% lower than in the untreated rebars. These findings support the feasibility of applying this treatment without compromising structural functionality.

## 1. Introduction

One of the primary causes of deterioration in reinforced concrete structures under service conditions is the corrosion of steel rebars, which leads to the degradation of both materials and may even reduce load-bearing capacity. As steel undergoes oxidation—a natural process in which it transforms into its most stable oxide—its volume increases due to the formation of corrosion products. This expansion induces stresses in the concrete cover, resulting in cracking and the failure of this protective layer due to tensile forces. Moreover, these surface alterations in the steel rebars can also affect the bond strength of the steel–concrete composite material [1].

Concrete, being an inherently alkaline material due to the presence of calcium hydroxide, alkalis, and other compounds in its composition, reaches a pH value close to 13 after manufacture, which influences its performance throughout its service life [2]. Consequently, according to the Pourbaix electrochemical equilibrium diagram for iron, rebars initially remain in a passive condition [3]. However, changes in the physicochemical environment of the steel, such as the presence of aggressive ions like sulphates or chlorides, can result in the loss of its initial passivation. Consequently, if thermodynamic conditions are favourable, the corrosion process will initiate when electrical currents with a sufficient potential difference facilitate the formation of a galvanic cell that triggers it [4,5,6]. In environments rich in these ions, corrosion onset occurs when the stability of the passive layer is compromised and the depassivation kinetics are altered. Once the steel transitions into an active corrosion phase, the use of inhibitors is one of the most widely adopted strategies to mitigate the corrosion damage [7,8].

Corrosion significantly impacts the bond strength of the composite material formed by steel and concrete. This phenomenon can occur through various mechanisms, either individually or in combination. These mechanisms include the following: (a) inducing the formation of corrosion products that, upon interacting with concrete, modify the mechanical and chemical properties of the bond zone; (b) reducing the height of the deformations on the rebars due to mass loss associated with the generated corrosion products; (c) causing cracks in the concrete, either due to the application of external loads or internal stresses resulting from the volumetric expansion of corrosion products; and (d) compromising the integrity of transverse reinforcement, such as stirrups or ties, thereby altering the confinement conditions of the concrete surrounding the rebars [9].

In recent decades, the use of cost-effective and environmentally friendly corrosion inhibitors has increased significantly [10]. Within this category, amino acids stand out due to their eco-friendly, non-toxic, biodegradable, and low-cost characteristics [11]. One of the most widely employed strategies to mitigate the kinetics of the corrosion process is the incorporation of inhibitors that facilitate the formation of protective monolayers on the metal surface, thereby reducing the rate of anodic or cathodic reactions. Furthermore, molecular self-assembly on the metal surface acts as an effective barrier, preventing the formation of layers composed of corrosion products [12]. In this field, numerous studies have focused on the composition of inhibitory monolayers, the procedures for their attachment to the steel surface, as well as their behaviour in the presence of aggressive ions that accelerate the kinetics of the corrosion process. However, no investigations have been identified that examine the effect of such monolayers on the bond between the steel rebar and the concrete, the potential variation in their inhibitory capacity due to the relative slip between both materials under dynamic loading, or the evolution of their properties under conditions simulating in-service structural behaviour.

The pull-out test has become one of the most used methods for studying the bond properties of the reinforcement-concrete system, and is extensively applied in various scientific studies [13,14]. This method enables the assessment of the system’s behaviour under different conditions, including the analysis of reinforcement deformation patterns, the evaluation of concrete cover, and the influence of confinement conditions [15]. Additionally, this technique is highly valuable for investigating the effect of corrosion on reinforcement and its impact on bond strength with concrete. Numerous studies have examined aspects related to the bonding of corroded rebars in concrete, the degradation of bond strength in reinforced concrete structures, and the behaviour of bond performance after fire exposure [16,17]. To date, no studies have been found that investigate, through pull-out tests, the mechanical behaviour of the steel–concrete system with an intermediate corrosion-inhibiting monolayer.

The formation of corrosion-inhibiting monolayers on the surface of rebars in rein-forced concrete structures requires a comprehensive analysis of their influence on the mechanical behaviour of the system, particularly regarding the bond strength between both materials. Furthermore, it is crucial to assess the evolution of the inhibitory capacity of these monolayers under pull-out testing, simulating the mechanical and environmental conditions that a structure experiences throughout its service life.

In this study, Scanning Electron Microscopy (SEM) and Energy-Dispersive X-ray Spectroscopy (EDX) were employed to characterise the morphology and elemental composition of the corrosion-inhibiting monolayers, providing detailed information on the compounds present on the reinforcement surface, both with and without the inhibitor treatment. Subsequently, pull-out tests were performed under cyclic loading conditions to evaluate the effects of these loads on both bond strength and the integrity of the monolayers. Finally, the corrosion kinetics of rebars were assessed by exposing them to a 0.5 M sodium chloride aggressive solution for one year.

The findings of this study will enable the establishment of correlations between the evolution of bond strength properties, the effectiveness of inhibitor monolayers under mechanical loading, and their resistance to aggressive environments. This will contribute to the development of more effective and sustainable strategies for the protection of reinforced concrete structures in harsh conditions.

## 2. Materials and Methods

### 2.1. Inhibitory Monolayers

Derived from inhibitory compounds such as amines or polycarboxylates, these substances adhere to the metallic surface through a chemical adsorption process, forming a protective organic layer. This layer acts as a barrier, reducing the kinetics of cathodic and anodic reactions, which significantly decreases the metal’s corrosion rate and consequently protects the reinforcement [18,19].

**Figure 1:**
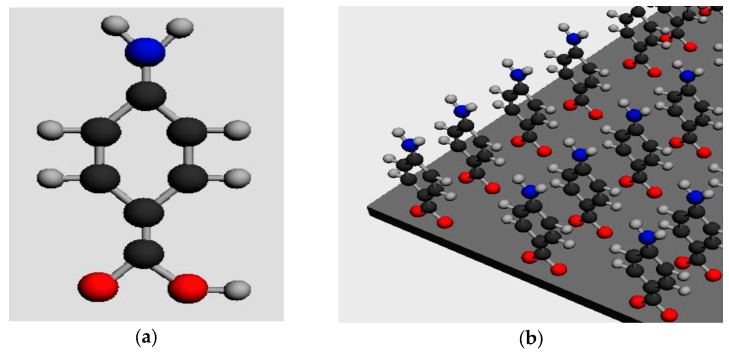
(**a**) Chemical structure of 4-aminobenzoic acid; (**b**) adhesion to the metal surface (red: oxygen; black: carbon; grey: hydrogen; blue: nitrogen).

In this study, the inhibitory monolayer was generated from 4-aminobenzoic acid (C_7_H_7_NO_2_), an aminocarboxylic compound that has demonstrated a remarkable ability to reduce corrosion kinetics [20,21,22]. The adhesion of the monolayer to the reinforcement occurs through chemical bonding, with interactions forming between the carboxyl group of the compound and the positive charges present on the metal surface (Figure 1).

To ensure its fixation to the steel rebar, two techniques commonly used in surface treatments of this nature were employed.

1. Natural binding. Prior to treatment, the surface of the rebars underwent a chemical pickling process by immersion in a 1 M phosphoric acid solution for 5 min. Subsequently, the rebars were rinsed with distilled water to ensure the complete removal of any residual pickling solution. Once prepared, the rebars were immersed in a solution of the active compound for 10 min. This method does not involve any additional intervention that could alter the natural chemical adhesion process, which is driven by the attraction between opposite charges, thereby facilitating the formation of chemical bonds between the inhibitory compound and the metal surface.
2. Electrolysis. Following the chemical pickling process and subsequent rinsing with distilled water, the rebars were immersed in the inhibitory solution. A constant current density of 0.5 ± 0.1 mA/cm^2^ was applied for 120 min using a regulated power supply unit (FAC-363B, Promax, Barcelona, Spain). Throughout the electrolysis process, the solution was maintained under constant agitation with a magnetic stirrer to ensure uniform monolayer formation. Additionally, a multimeter connected in series was incorporated to monitor and adjust the applied potential, ensuring current stability throughout the process.

### 2.2. Reinforced Concrete Specimens

**Figure 2:**
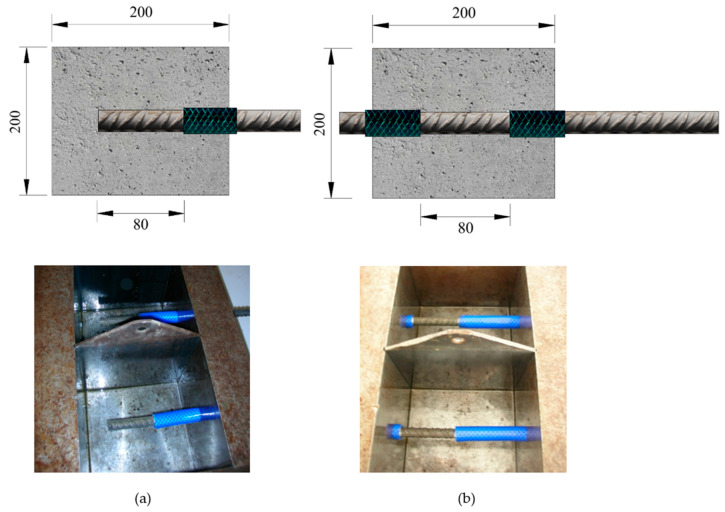
(**a**) Cubic specimens for corrosion studies; (**b**) cubic specimens for bond strength studies (measurements in mm).

A single type of reinforced concrete specimens was manufactured for the two tests considered in this study: bond strength and corrosion analysis. In 200 mm cubic specimens, rebars with a diameter of 16 mm, made of B 500 SD steel, were embedded in each specimen. The rebars, measuring 700 mm in length, were positioned perpendicular to the casting direction. The anchorage length was 80 mm (5Ø), while the remaining portion of the rebar inside the specimen was protected from direct contact with the concrete using a rubber sleeve (Figure 2). For each of the three groups of rebars under study (natural binding, electrolysis, and no treatment), a total of 16 specimens were produced: 8 for the bond strength test and 8 for the corrosion rate test.

In the specimens specifically designed for the analysis of corrosion processes, one end of the rebar was completely embedded in the concrete to ensure a uniform concrete cover on all sides of the specimen (Figure 2a). Conversely, in the specimens intended for bond strength tests, the rebars extended from both ends of the concrete cubes (Figure 2b).

The concrete used in this study was prepared with CEM II/B-M 42.5R cement and crushed limestone aggregates: 4/12 gravel and 0.125/4 sand. The admixture employed was a polycarboxylate-based superplasticizer (Viscocrete 3425). The characteristics of the mix are presented in Table 1.

All specimens were demoulded 24 h after casting and stored in a curing chamber at 20 °C with a relative humidity above 95% until they reached the required age for testing.

During the manufacturing process of the cubic specimens, cylindrical specimens measuring 150 mm in diameter and 300 mm in height were also prepared to perform compressive strength tests on the concrete at 28 days [23]. Two cylindrical specimens were prepared for each of the three rebar treatments. The results of these tests are presented in Table 2. The abbreviations used to identify each batch correspond to the treatment applied to the rebar: NT (no treatment), T-NB (natural binding), and T-EL (electrolysis).

Compressive strength is a factor that significantly influences the steel–concrete bond and the corrosion of reinforcements. Therefore, the differences recorded among the three batches may affect the results obtained in these tests. To reduce this influence, the normalised bond strength was determined in the bond strength tests (see Section 3.2).

A summary detailing all tested specimens and their key characteristics is given in Table 3.

### 2.3. Methods

#### 2.3.1. Scanning Electron Microscopy Tests (SEM) and Energy Dispersive X-Ray Spectroscopy (EDX)

In order to assess the adhesion capacity of the inhibitor monolayer to steel, the surface of the steel was analysed on rebars that had previously been subjected to a significant state of stress, with the consequent deterioration of the steel–concrete interfacial zone. For this purpose, samples were taken from the rebars used in the pull-out tests to failure (Section 2.3.2) after the completion of these tests. Three samples were prepared to analyse the existing elements on the steel surface: non-treated steel (NT), inhibitory monolayer fixed through natural binding (T-NB), and inhibitory monolayer fixed via electrolysis (T-EL). The surface of each sample was studied using a scanning electron microscope (JEOL JSM-6300, JEOL Ltd., Akishima, Tokyo, Japan) and a field emission scanning electron microscope (Ultra 55, Carl Zeiss AG, Oberkochen, Germany), both equipped with an energy dispersive X-ray (EDX) spectrometer for phase composition analysis.

#### 2.3.2. Bond Strength Test

The application of a surface treatment on the reinforcement can negatively affect the steel–concrete bond, which is the basic phenomenon on which the performance of reinforced concrete as a structural material is based. To analyse the bond strength of the rebars treated with the corrosion-inhibiting layer, pull-out tests were conducted on 200 mm cubic concrete specimens in accordance with European Standard EN 10080:2006 [24].

The pull-out load was applied progressively up to bond failure and the relative slip of the rebar was measured using two linear variable differential transformers (LVDTs) connected to the unloaded end of the rebar. The bond stress (τ) was calculated using Equation (1):

(1) $$\tau =\frac{F}{\pi \;Ø\;L}$$

where F is the applied load, Ø the reinforcement rebar’s nominal diameter, and L is the effective anchorage length.

#### 2.3.3. Corrosion Test

Before initiating the corrosion monitoring process, the specimens were subjected to a loading procedure through pull-out tests to simulate the stresses experienced by reinforcement under service conditions in a real structure. These stresses could compromise the integrity of the inhibitory coating, potentially reducing its effectiveness as a corrosion barrier. For this purpose, a cyclic load consisting of 1000 load–unload cycles was applied. According to the literature reviewed, the applied load ranges vary among different authors [25,26,27]. Considering the stresses typically experienced by reinforcement in a properly designed structure, this study adopted a load range between 25% and 50% of the ultimate failure load. According to the results obtained in the bond strength tests (Section 2.3.2), the maximum load reached was approximately 80 kN; therefore, the lower and upper load limits were set at 20 kN and 40 kN, respectively. The tests were conducted on 200 mm cubic concrete specimens.

After the pull-out tests, the specimens were partially submerged (up to mid-height) in a 0.5 M sodium chloride solution and left under these conditions for one year to simulate a very aggressive environment (XS3 environment). The rebar end was protected with vaseline in order to prevent corrosion. The corrosion study was conducted by measuring the evolution of the corrosion rate over time. This parameter provides quantitative information about the reinforcement corrosion kinetics. The corrosion rate measurements allow the calculation of the rebar section loss per time unit, expressed in μm/year assuming that homogeneous corrosion occurs.

The most common techniques to monitor the corrosion rate are electrochemical techniques, which consist of the polarization of the rebar by forcing a small electrical current. A voltage is applied, and the response current is measured. The application of this technique provides the density of corrosion current (i_CORR_), which is the electrical current per unit surface area of the rebar onto which the corrosion processes are taking place.

To calculate this parameter, the Linear Polarization Resistance technique was applied [28], considering the ohmic drop between the rebar and concrete surface. Once i_CORR_ was determined, the following Equation (2), derived from Faraday’s law, was used to calculate the corrosion rate (V_CORR_), which represents the rebar radius loss per unit time and is proportional to the loss of metal per unit surface area per unit time:

(2) $$v_{corr}=3.27\frac{i_{corr}\cdot M}{n\cdot \rho }=11.6\;i_{corr}$$

where V_CORR_ corresponds to the corrosion rate (μm/year), i_CORR_ is the corrosion current density (μA/cm^2^), M represents the atomic mass of steel (g/mole), ρ is the steel density (g/cm^3^), and n denotes the number of electrons in the reaction (2 in the case of steel).

The corrosion current density measurements were taken using a three-electrode cell, where the embedded rebar served as the working electrode. The reference electrode used was a saturated calomel electrode, while a stainless-steel mesh acted as the counter electrode, positioned around the specimen to make the electric field as homogeneous as possible. A wet cloth was placed between the concrete surface and the counter electrode to ensure good electrical contact. A potentiostat (PGSTAT 100, Metrohm Autolab, Utrecht, The Netherlands) was employed for the measurements. To reduce electrical noise, tests were conducted in a Faraday cage. Eight groups of specimens were manufactured per treatment type for the corrosion tests following cyclic loading. The arithmetic mean of the obtained values was taken as the test result.

## 3. Results and Discussion

### 3.1. Scanning Electron Microscopy (SEM) and Energy Dispersive X-ray Spectroscopy (EDX)

**Figure 3:**
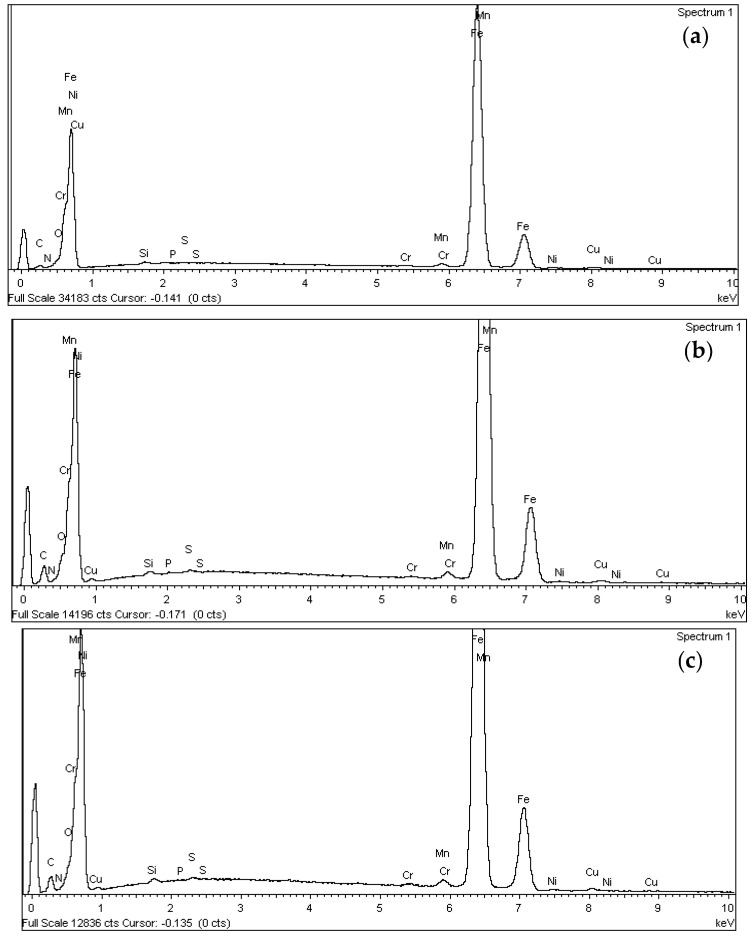
EDX spectrogram and the chemical composition. (**a**) untreated rebar; (**b**) rebar with monolayer applied by natural binding; (**c**) rebar with monolayer applied by electrolysis.

After performing the pull-out tests to failure (Section 3.2) and thus proceeding to the complete deterioration of the steel–concrete interfacial zone, the surface of the rebar was analysed to determine whether the inhibitor monolayer remained adhered to the steel. Figure 3 shows the energy dispersive X-ray (EDX) spectrogram of the non-treated and treated rebar with the 4-aminobenzoic acid monolayer applied by natural binding and electrolysis.

The component spectrum is very similar in all three cases, as 4-aminobenzoic acid consists of carbon, oxygen, and nitrogen, in addition to hydrogen elements already present in the steel. Furthermore, a notable similarity is observed in the spectra of the rebar coated with the inhibitor through natural binding and electrolysis (Figure 3b,c), which is expected since the same compound was applied in both cases.

These differences are particularly evident in the relative concentrations of oxygen and nitrogen, which tend to increase in the specimens treated with the inhibitory monolayer. This suggests that the surface modification was successful, at least in terms of the chemical presence of the inhibitor. Overall, while the general spectral profiles remain comparable, the elemental distribution confirms that applying the 4-aminobenzoic acid monolayer—either by natural binding or electrolysis—induces detectable changes in the surface chemistry of the steel reinforcement. In terms of elemental composition, certain differences can be observed between the non-treated rebars and those with inhibitory monolayers.

Table 4 shows the surface chemical composition, where weight (%) refers to the mass proportion of each element, and mole fraction represents the atomic proportion based on the number of atoms present.

An analysis of the quantitative data on the weight percentage of each element under the different treatment conditions allows for the following conclusions to be drawn.

1. The iron (Fe) percentage decreases from 93.87% in non-treated rebar to an average of 80% after the application of the treatment. This result is particularly significant as it suggests a reduction in the exposed steel surface following treatment. Additionally, the values obtained for both techniques are very similar, indicating a comparable distribution of the inhibitory compound on the steel surface.
2. The oxygen (O) content on the surface increases from 0.81% to values above 5%. This increase is attributed to the inhibitory compound, which introduces oxygen through the carboxyl group. This behaviour confirms the presence of the compound on the metal surface, demonstrating its effective adsorption.
3. The presence of carbon (C) on the surface increases from 3.22% to values close to 12%, representing, along with iron (Fe), the most significant variation observed. This increase can be attributed to 4-aminobenzoic acid, which consists of an aromatic ring primarily composed of carbon and hydrogen, characteristic elements of organic compounds. This variation further reinforces the evidence of the compound’s presence on the metal surface.
4. Regarding nitrogen (N), an element contributed by the amino group of the inhibitory compound, its percentage increases from 0.01% to an average value of 0.80%. This increase provides further evidence of the presence and distribution of the compound on the steel surface.
5. No significant differences were observed in the content of the remaining analysed components.

### 3.2. Bond Strength

The analysis of the results has been carried out taking into account the fact that the concrete–steel bond depends to a large extent on the quality of the concrete and primarily on its compressive and tensile strength. ACI Code 318 [29] and FIB Bulletin No. 1 [30] relate bond stress to the square root of the compressive strength. Therefore, to enable comparison of the bond strength of reinforcement embedded in concrete of different qualities (Table 5), the normalised bond strength (τ_n_) has been determined using Equation (3), which is the criterion most frequently found in the literature:

(3) $$\tau_{n}=\frac{\tau }{\sqrt{f_{c}}}$$

where τ is the bond strength, and f_c_ the compressive strength of the batch tested.

The test results are listed in Table 5, along with the mode of failure.

The analysis of variance (ANOVA) of the results reveals no statistically significant differences between the three study groups (*p*-value = 0.56), considering a significance level of 5%. Consequently, it can be concluded that the formation of inhibitory monolayers using both techniques does not affect the bond strength between reinforcement and concrete, exhibiting behaviour similar to that of untreated specimens.

**Figure 4:**
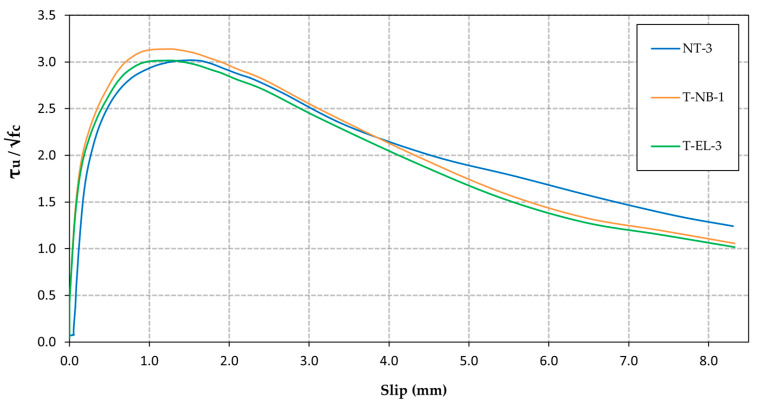
Curves of normalised bond stress versus slip corresponding to specimens NT-3 (no treatment), T-NB-1 (natural binding), and T-EL-3 (electrolysis).

As an example, Figure 4 shows the normalised bond stress vs. slip curves corresponding to 3 of the 24 specimens tested (NT-3, T-NB-1, and T-EL-3), one from each of the three treatment groups analysed. The three studied groups exhibit very similar bond behaviour. Initially, the bond strength increases with slip due to mechanical anchorage and chemical interaction at the interface. Subsequently, the bond stress reaches its peak, at which point the rebar is fully anchored, and force transfer is at its maximum. Finally, pull-out failure occurs, and bond strength begins to decrease due to progressive damage at the interface between the rebar and the concrete, leading to bond failure, rebar slip, and the loss of load transfer capacity.

### 3.3. Corrosion Rate

Under service conditions, reinforcement deformation and friction between the rebar and the concrete, caused by the relative slip between both materials, may lead to localised damage in the inhibitory monolayer. As a result, the applied treatment could lose its effectiveness, promoting the formation of localised corrosion anodes in the affected areas. To address this issue, the corrosion rate was measured at different ageing stages in specimens subjected to loading. After 1000 load–unload cycles, the specimens were partially submerged in a 0.5 M sodium chloride solution, simulating sea water conditions to assess their corrosion resistance for one year.

Considering the obtained corrosion values, the mass loss (Δm) was calculated by integrating the curve of i_CORR_ (μA/cm^2^) values obtained during the monitoring process, in accordance with Faraday’s law (Equation (4)).

(4) $$\Delta m=\frac{M}{n\;F}\cdot \int_{t_{0}}^{t}i_{CORR}\cdot dt$$

where M is the atomic mass of steel (55.845 g/mol), t is the time in seconds, n is the number of electrons released or acquired during the corrosion process (2 for this case) and F is Faraday’s constant (96845 C/mol). The mass loss (Δm) was expressed in mg/cm^2^.

**Figure 5:**
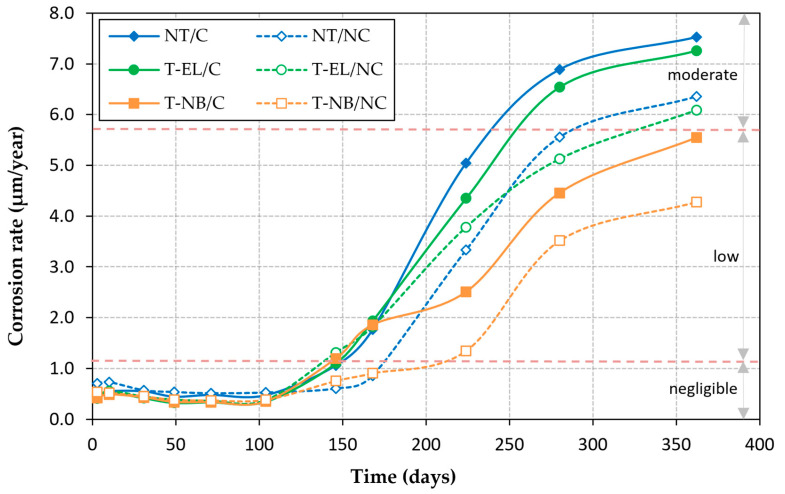
Evolution of corrosion rate (C: rebars previously subjected to 1000 load–unload cycles; NC: rebars that have not been subjected to a loading process).

**Figure 6:**
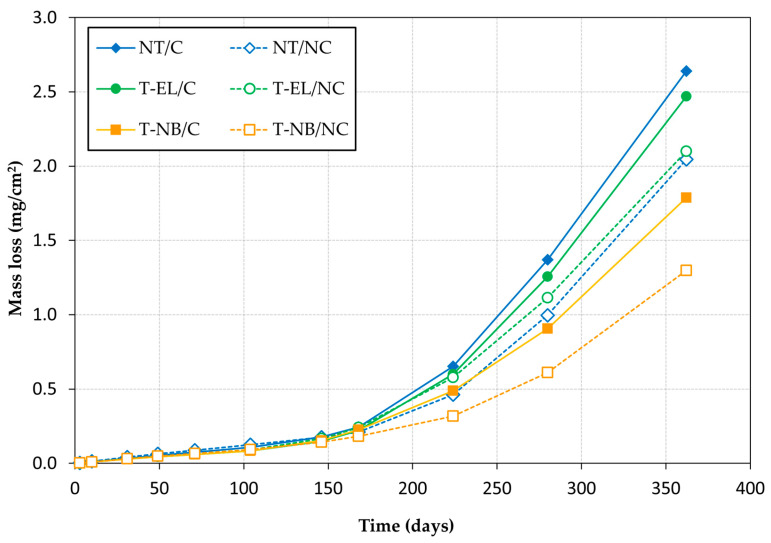
Evolution of the mass loss of steel rebars (C: rebars previously subjected to 1000 load-unload cycles; NC: rebars that have not been subjected to a loading process).

Figure 5 and Figure 6 show the evolution of the average V_CORR_ and the loss of steel mass in the specimens, where C refers to the rebars that have previously undergone 1000 load-unload cycles, and NC refers to those that have not undergone a loading process. Figure 5 also includes the thresholds considered in Standard UNE 112072:2011 [31], ASTM STP 1065 [32], and RILEM TC-154 [33], corresponding to the different corrosion levels: negligible (v_CORR_ < 1.1 µm/year), low (1.1 µm/year < v_CORR_ < 5.8 µm/year), moderate (5.8 µm/year < v_CORR_ < 11.6 µm/year), and high (v_CORR_ > 11.6 µm/year).

In all cases, the rebar corrosion rate tended to increase approximately five months after the specimens were partially submerged in the chloride solution. This suggests that chlorides began penetrating the concrete, reaching the reinforcement and breaking down the passive oxide layer. When comparing the different sample groups, the following conclusions can be drawn:

(a) Influence of loading

In both non-treated reinforcement rebars (NT) and those treated with inhibitory monolayers (T-NB and T-EL), an increase in the corrosion rate was observed in the rebars subjected to cyclic loading. After one year, this increase was 18% in NT rebars, 29% in T-NB rebars, and 19% in T-EL rebars. These results suggest that the loading process adversely affects the passive layer that protects the reinforcement from corrosion, accelerating corrosion kinetics. Consequently, steel mass loss in the rebars subjected to cyclic loading was 29%, 38%, and 18% greater in NT, T-NB, and T-EL rebars, respectively.

(b) Influence of inhibitor treatment

The rebars treated with the inhibitor exhibited less corrosion than the non-treated rebars. This effect is particularly significant in the rebars treated with the natural fixation method of the inhibitor monolayer (T-NB), where, after one year of testing, the steel section loss was 32% lower than in the untreated rebars for the loaded specimens, and 37% lower for the unloaded specimens. The treatment was less effective when the inhibitor monolayer was applied by electrolysis (T-EL). In this case, the steel section loss was only 6% lower than in the untreated rebars for the loaded specimens. However, this lower efficacy may be influenced by the lower compressive strength of the concrete used in the T-EL specimens (Table 2), as the resistance to chloride penetration into the material largely depends on the quality of the concrete.

The efficacy of the inhibitor monolayer confirms the results obtained in SEM and EDX tests (Section 3.1), where it was observed that in rebars that have been subjected to significant stresses, the monolayer remains adhered to the steel surface and therefore continues to protect it against corrosion.

## 4. Conclusions

The main results obtained from the combined study of pull-out tests and electrochemical analysis are as follows:

1. The Scanning Electron Microscopy (SEM) tests show that the exposed steel surface decreases after the application of the inhibitor monolayer. The distribution of this layer on the steel surface is similar for both techniques analysed in the study: natural binding and electrolysis.
2. The application of the inhibitor monolayer by natural binding or electrolysis does not affect the bond strength between reinforcement and concrete, exhibiting behaviour similar to that of the untreated rebars.
3. The application of load–unload cycles adversely affects the passive layer that protects the reinforcement from corrosion, accelerating corrosion kinetics. In specimens submerged in seawater for one year, the steel mass loss in rebars that had previously been subjected to cyclic loading was higher than in those that had not: 18% in NT rebars, 29% in T-NB rebars, and 19% in T-EL rebars.
4. The rebars treated with the inhibitor exhibited less corrosion than the untreated rebars. This reduction in corrosion is particularly significant in the rebars treated with the natural fixation method of the inhibitor monolayer, where, after one year of testing, the steel section loss was 32% lower than in the untreated rebars for the loaded specimens and 37% lower for the unloaded specimens.
5. The inhibitor monolayer provides less corrosion protection when the treatment is applied by electrolysis than when it is applied by natural binding. However, since in each case the concrete strength of the test specimens was not the same, it is not possible to attribute this poorer performance solely to the treatment application technique, and further research is needed to determine the real impact of the application method.

In this research work, the behaviour of inhibitor monolayers against corrosion was analysed under simulated real-service conditions of structures. However, much longer-term studies are needed to truly assess the efficacy of these treatments. Furthermore, the study should be extended to prestressed elements, where the bars are subjected to very high stresses and strains that may damage the superficially applied monolayer. Finally, this study is also expected to serve as a basis for future research exploring monolayers composed of alternative eco-friendly organic compounds.

## Figures and Tables

**Table 1:** Mixture proportions of concrete.

Cement (kg/m^3^)	Water (kg/m^3^)	w/c	Admixture (kg/m^3^)	Sand (kg/m^3^)	Gravel (kg/m^3^)
340.0	187.0	0.55	2.7	1,108.3	738.8

**Table 2:** Results of compressive strength test (MPa).

Batch	Specimen 1	Specimen 2	Mean
NT	51.51	52.70	52.11
T-NB	48.09	49.99	49.04
T-EL	43.71	46.79	45.25

NT: no treatment; T-NB: natural binding; T-EL: electrolysis.

**Table 3:** Tested specimens.

No. Batches	Test					
	Bond (Pull-out)		Corrosion Rate		Compressive Strength	
	Specimen	No. Specimens	Specimen	No. Specimens	Specimen	No. Specimens
3 (1 per treatment)	Cubic (Figure 2a) (200 mm)	24 (8 per batch)	Cubic (Figure 2b) (200 mm)	24 (8 per batch)	Cylindrical (∅150 mm)	6 (2 per batch)

**Table 4:** Chemical composition of the steel surface for each treatment.

Element	No Treatment		Natural Binding		Electrolysis	
	Weight (%)	Mole Fraction	Weight (%)	Mole Fraction	Weight (%)	Mole Fraction
Fe	93.87	0.8223	80.08	0.5055	79.91	0.5005
C	3.22	0.1311	11.53	0.3384	11.93	0.3475
Mn	0.86	0.0076	0.93	0.0060	0.84	0.0053
O	0.81	0.0248	5.52	0.1216	5.33	0.1165
Cu	0.46	0.0036	0.46	0.0026	0.47	0.0026
Si	0.31	0.0053	0.28	0.0035	0.27	0.0033
Ni	0.19	0.0016	0.11	0.0007	0.15	0.0009
Cr	0.14	0.0013	0.16	0.0011	0.11	0.0007
P	0.10	0.0016	0.13	0.0015	0.12	0.0013
S	0.04	0.0006	0.06	0.0015	0.04	0.0004
N	0.01	0.0002	0.74	0.0186	0.84	0.0210
Total	100	1	100	1	100	1

**Table 5:** Mechanical properties and pull-out test results in 200 mm cubic specimens.

Rebar Treatment	Batch	Specimen	Maximum Load (kN)	τ_u_ (MPa)	f_c_ (MPa)	τufc	Mode of Failure
No treatment	NT	NT-1	84.12	20.92		2.90	Pull-out
	NT	NT-2	90.64	22.54		3.12	Pull-out
	NT	NT-3	87.80	21.83		3.02	Pull-out
	NT	NT-4	88.33	21.97	52.11	3.04	Pull-out
	NT	NT-5	84.33	20.97		2.91	Pull-out
	NT	NT-6	79.68	19.81		2.74	Pull-out
	NT	NT-7	88.43	21.99		3.05	Pull-out
	NT	NT-8	102.15	25.40		3.52	Pull-out
Natural binding	T-NB	T-NB-1	87.45	21.75		3.11	Pull-out
	T-NB	T-NB-2	87.42	21.74		3.10	Pull-out
	T-NB	T-NB-3	82.89	20.61		2.94	Pull-out
	T-NB	T-NB-4	89.25	22.19	49.04	3.17	Pull-out
	T-NB	T-NB-5	81.33	20.23		2.89	Pull-out
	T-NB	T-NB-6	78.57	19.54		2.79	Pull-out
	T-NB	T-NB-7	83.63	20.80		2.97	Pull-out
	T-NB	T-NB-8	73.81	18.36		2.62	Pull-out
Electrolysis	T-EL	T-EL-1	84.77	21.08		3.13	Pull-out
	T-EL	T-EL-2	75.15	18.69		2.78	Pull-out
	T-EL	T-EL-3	80.54	20.03		2.98	Pull-out
	T-EL	T-EL-4	83.49	20.76	45.25	3.09	Pull-out
	T-EL	T-EL-5	83.75	20.83		3.10	Pull-out
	T-EL	T-EL-6	71.34	17.74		2.64	Pull-out
	T-EL	T-EL-7	82.70	20.57		3.06	Pull-out
	T-EL	T-EL-8	72.91	18.13		2.70	Pull-out

## Data Availability

The original contributions presented in this study are included in the article. Further inquiries can be directed to the corresponding author.
